# Can residual kidney function affect quality of life and cognitive function in hemodialysis patients?

**DOI:** 10.1186/s12882-022-02892-7

**Published:** 2022-07-23

**Authors:** Asmaa Elgendy, Adel I. Abdelsalam, Mostafa Mansour, Mohammed K. Nassar

**Affiliations:** 1 Mansoura Nephrology and Dialysis Unit, Internal Medicine Department, Mansoura University, Mansoura, Egypt; 2 Rheumatology and Immunology Unit, Internal Medicine Department, Mansoura University, Mansoura, Egypt; 3 Clinical Pathology Department, Mansoura faculty of medicine, Mansoura, Egypt

**Keywords:** Chronic kidney disease, Hemodialysis, Residual kidney function, Quality of life, Cognitive function

## Abstract

**Background:**

Residual kidney function (RKF) may provide many benefits to patients on permanent renal replacement therapy that are reflected in better control of biochemical parameters. In hemodialysis patients, quality of life (QOL) and cognitive function are often impaired. This study aimed to assess the predictors of RKF and its impact on QOL and cognitive function in chronic hemodialysis patients.

**Patients and methods:**

The study involved seventy-eight patients suffering from end-stage renal disease on regular hemodialysis. The patients were divided into two groups according to the presence or absence of RKF (24-hour urine volume ≥ 100 ml). Beside basic laboratory investigations, all patients were subjected to Kidney Disease Quality of Life-Short Form (KDQOL-SF) version 1.3 for assessing the quality of life and Montreal cognitive assessment (MoCA) score for assessing cognitive function.

**Results:**

There was a significantly higher score for KDQOL domains and MoCA score in patients with RKF compared to patients without RKF. There was a significant positive correlation between RKF and both of MoCA score and the physical composite score (PCS) of QOL. Moreover, there were statistically significant positive correlations between the MoCA score and both PCS and mental composite score (MCS). On multivariate analysis, hemodialysis duration was the only predictor for RKF; whereas age was a significant predictor for PCS; and MoCA score could be significantly predicted by the measured RKF and patients’ age.

**Conclusion:**

HD patients with maintained RKF had better QOL and cognitive function. The duration of HD and the age of the patients were found to be related to RKF and PCS in this study. RKF was associated with the cognitive performance of hemodialysis patients.

## Background

Health-related quality of life (HRQOL) is a multidimensional index to calculate wellbeing. It is related to the physical, emotional, mental, and social functioning of people or cases [1]. Dialysis patients have compromised HRQOL when compared with the general population and have decrements comparable to cases with other chronic diseases including cancer and heart failure [2]. HRQOL might be influenced by a lot of factors, such as clinical manifestations, adverse effects of medications, nutritional condition, hospitalization, and certain biochemical factors involving Kt/V, calcium-phosphorus (Ca × P) product, parathyroid hormone (PTH) levels, anemia, and serum albumin level [3]. HRQOL might enhance over time as cases grow accustomed to the HD therapy, however, parameters including recurrent hospital admission, adverse effects and related medications, as well as RKF loss might induce HRQOL worsening [4].

Cognitive impairment (CI) is described as recently appearing deficits in two or more regions of cognitive functions, such as memory deficits, executive functioning, attention or speed of data processing, or language [5]. Cognitive impairment and dementia are more prevalent in ESKD patients who receive hemodialysis compared with age matched general population [6]. Patients with CI are at higher risk of hospitalization, mortality, and a poorer quality of life [7]. Furthermore, CI in patients with ESKD may reduce their abilities to adhere to complex medical or dietary regimens and to fully participate in medical decisions [8].

RKF is the remaining kidney function in cases receiving renal replacement therapy (RRT) for renal failure [9]*.* Higher RKF is associated with better outcomes such as improved survival by maintaining fluid and metabolic homeostasis, mitigating mineral abnormalities, optimizing uremic toxin clearance, and sustaining higher production of endogenous vitamin D and erythropoietin [10]. RKF is recognized as an important factor influencing morbidity, mortality, and quality of life in chronic dialysis patients [11].

Even though higher RKF is associated with better results, RKF is not often assessed or taken into consideration when determining clinical care or dialysis prescription in HD practice. Therefore, this study was carried out to assess the impact of RKF on quality of life and cognitive function in chronic HD patients and to evaluate clinical and laboratory findings of such patients as predictors of RKF.

## Methods

In this cross-sectional observational study, seventy-eight adult (age > 18) ESRD patients undergoing regular hemodialysis for more than 6 months in the Dialysis Unit of Mansoura University Hospitals were recruited. Patients with neurological deficits and recent cerebrovascular accidents within the previous 6 months, taking medications known to affect the cognitive status (e.g. psychoactive drugs, antidepressants and anticonvulsants) or suffering from decompensated organ failure, other than renal failure (e.g. decompensated hepatic or heart failure), were excluded. The sample size was selected as a convenient sample; all patients who fulfilled the inclusion criteria were offered to participate in the study unless they qualified for any of the exclusion criteria or refused to participate. The study protocol was approved by the Institutional Research Board of the Faculty of Medicine, Mansoura University (approval registration number: MS.18.12.403). The study was explained to all patients and informed written consent was obtained from all of them before starting the study.

The patients were divided into two groups; group A (with RKF) (*n* = 29) who pass ≥100 ml/day of urine and group B (no RKF) (*n* = 49) who pass urine volume < 100 ml/day [12]. All patients were subjected to pre-dialysis basic laboratory investigations (complete blood count (CBC), serum iron, total iron binding capacity (TIBC), transferrin saturation (TSAT), serum ferritin, serum calcium, phosphorus, PTH) on the first session of the week in addition to RKF measurement, quality of life and cognitive function assessment.

### Residual kidney function measurement

**Fig. 1:**
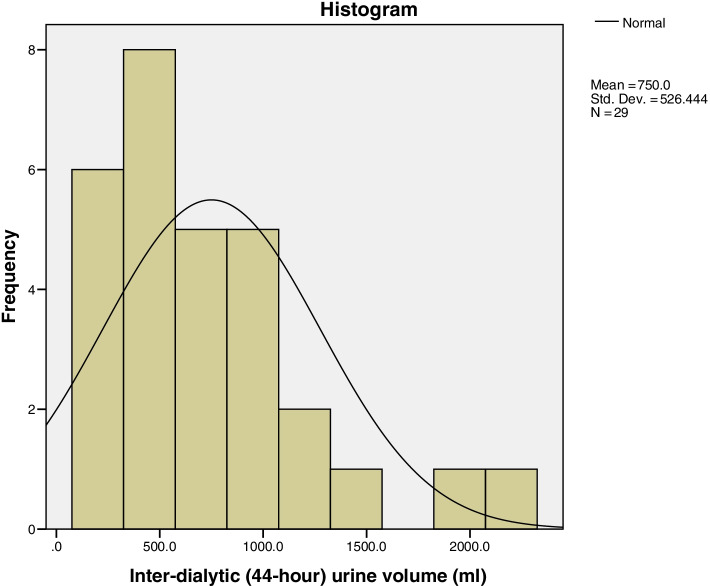
Histogram showing distribution of interdialytic (44-hour) urine output (ml) in patients with Residual Kidney Function who had Urine Output ≥100 ml / day (n: 29)

As aforementioned, RKF, in the current study, was identified if the patient passes ≥100 ml urine/day. Blood samples for measurement of blood urea nitrogen (BUN) were collected at the end of the first dialysis session of the week (BUN1) and immediately before the next session (BUN 2). Between these blood samples, urine was collected throughout the interdialytic period (44-hour urine collection). Figure 1 shows a histogram of estimated inter-dialytic (44-hour) urine volume. Then residual renal function was measured using this eq. [13]:

- Interdialytic urine volume x urine urea concentration / interdialytic period / mean blood urea nitrogen.

Where mean blood urea nitrogen = (BUN 1 + BUN2)/2.

Despite underestimating inulin clearance by about 20%, urea clearance (KRU) is not largely affected by dialysis conditions and is recommended, either alone or with creatinine clearance and taking the mean of both, by the European guidelines [14] and NKF-K/DOQI guidelines [15].

### Quality of life assessment

The validated Kidney Disease Quality of Life-Short Form (KDQOL-SF version 1.3) was used to assess HRQOL [16]. KDQOL-SF 36 was issued by *Hays* et al. *in 1994* and is published for free on internet website: http://gim.med.ucla.edu/kdqol/downloads-download.html. The KDQOL-SF is divided into a generic part and a disease-specific part. First, the generic part is formed by the SF-36 and consists of 36 questions measuring eight multi-item measures of physical and mental health status (physical functioning, role limitations caused by physical health problems, bodily pain, general health, vitality, social functioning, role limitations caused by physical health problems and mental health). Results from the SF-36 instrument are further summarized into a physical composite summary (PCS) score and a mental composite summary (MCS) score [17]. These summaries are constructed so that a score of 50 represents the mean of the general United States population with a standard deviation of 10 [18]. Second, the disease-specific part of the KDQOL-SF consists of 44 kidney disease-targeted questions; symptom/problem list (12 items), effects of kidney disease (8 items), burden of kidney disease (4 items), work status (2 items), cognitive function (3 items), quality of social interaction (3 items), sexual function (2 items), sleep (4 items), social support (2 items), patient satisfaction (1 item), and dialysis staff encouragement (2 items). In each domain, the responses receive a score from 0 to 100. Higher scores in the KDQOL and individual sub-sections of the survey reflect a better quality of life [19].

An Arabic translation of the KDQOL-SF version 1.3 was used in a prior research in Alexandria, Egypt [20], and the authors concluded that this translation is a validated and reliable instrument for assessing HRQOL in ESRD patients. This Arabic version of the KDQOL-SF1.3 was also used in the current study for HRQOL evaluation, with some extra parts translated from the original English form to suit patients on regular hemodialysis.

### Cognitive function assessment

Cognitive Function was assessed with the Montreal Cognitive Assessment (MoCA) test. The MoCA test is a 30-point test to assess several cognitive domains: Visuospatial/executive, Naming, Short term Memory, Attention, Language, Abstraction and Temporospatial orientation. MoCA score has been translated and adapted into several languages and is available freely on the Internet (available at www.MoCAtest.org). MoCA score ranges between 0 and 30 with a score of 26 and higher generally considered normal [21].

### Statistical analysis

SPSS (Statistical Package of Social Sciences) version 21 for Windows (SPSS, Inc) was used to conduct the statistical analysis. Number and percent were used to describe qualitative data (n, %). When applicable, the data was first evaluated for normality using the Shapiro-Wilk test or the Kolmogorov-Smirnov test. For normally distributed data, mean ± SD (standard deviation) was used, and for non- normally distributed data, median (interquartile range) was used. When comparing two groups with quantitative normally distributed data, the Independent Samples t-test was used, whereas when comparing two groups with quantitative non- normally distributed data, the Mann-Whitney test was employed. When comparing qualitative data with a 2 × 2 table, the chi-square or Fisher’s exact test was applied. Univariate correlation analysis with the Pearson test for normally distributed data and the Spearman test for non-normally distributed variables. The entry strategy was used to do multiple linear regression analysis. The model took into account the variables that can possibly influence the measured GFR, PCS score, and overall MoCA score. Variables were chosen for their statistical significance between the two groups, as well as relevant sociodemographic and clinical factors (age, gender, diabetes mellitus and hypertension). The R-squared test was used to see how much variation the model explained. χ2 goodness of fit tests were used to evaluate the model’s goodness of fit. A statistically significant *P* value was less than 0.05.

## Results

Group A (urine output ≥100 mL/day) patients had a significantly lower dialysis vintage and percentage of patients with positive HCV antibodies (*p* < 0.001 and 0.004, respectively), while there was no statistically significant difference between the two groups regarding the prevalence of diabetes mellitus or hypertension nor the different laboratory results. (Table 1). Unfortunately, only twenty-nine patients constituting more than a third of the examined patients pass appreciable urine volume and have measurable residual kidney function (Table 2). The median RKF measurement was 0.68 ml/min with interquartile range between 0.3 and 1.52 ml/min.

**Table 1:** Demographic, clinical and laboratory data according to residual renal function

	RKF (yes) *n* = 29 (≥100 mL/day)	RKF (No) *n* = 49 (> 100 ml/day)	*P*
Demographic and clinical data			
Age (years)	45 (33–63)	57 (42–65)	0.127
Gender / male	16 (55.2%)	30 (61.2)	0.599
Diabetes mellitus	3 (10.3%)	7 (14.3%)	0.736
Hypertension	26 (89.7%)	39 (79.6%)	0.351
Original kidney disease:			0.39
Hypertensive nephrosclerosis	11 (37.9%)	22 (44.9%)	
Diabetic nephropathy	3 (10.3%)	6 (12.2%)	
Polycystic kidney disease	0	2 (4.1%)	
Obstructive uropathy	0	3 (6.1%)	
Hereditary	0	2 (4.1%)	
Reflux nephropathy	1 (3.4%)	0	
Lupus nephritis	2 (6.9%)	0	
Chronic glomerulonephritis	1 (3.4%)	0	
Failed kidney transplant	0	1 (2%)	
Chronic interstitial nephritis	4 (13.8%)	0	
Unknown	6 (20.7%)	13 (26.5%)	
Drug-induced	1 (3.4%)	0	
HCV positivity	1 (3.4%)	15 (30.6%)	**0.004**
Parathyroidectomy	0	3 (6.1%)	NA
HD duration (years)	2 (1.5–3)	6 (4–8)	**< 0.001**
BMI (Kg/m^2^)	30.1 (24.9–34.4)	27.1 (24.9–32.9)	**0.22**
Laboratory data			
Hemoglobin (g/dl)	10.6 (9.45–11.5)	10.7 (9.9–11.8)	0.498
TSAT (%)	28 (21–38.5)	31 (22.5–39.5)	0.441
Ferritin (ng/ml)	543 (285–927)	552 (315–1037.5)	0.714
Calcium (mg/dl)	8.8 (8–9.19)	8.8 (7.85–9.19)	0.796
Phosphorus (mg/dl)	5.7689 ± 2.0651	5.6326 ± 1.79691	0.760
PTH (pg/ml)	483 (277.5–884.5)	736 (379.5–1047.5)	0.141
KT/V	1.1547 (0.8567–1.3402)	1.2563 (0.86–1.6301)	0.329

*Abbreviation*: *HCV* Hepatitis C virus, *HD* Hemodialysis, *TSAT* Transferrin saturation, *PTH* Parathyroid hormone

**Table 2:** Characteristics of RKF (≥100 ml/day)

Variables	Frequency
**RKF:**	
**Yes (≥100 ml/day)**	29 (37.2%)
**No (> 100 ml/day)**	49 (62.8%)
**KRU measurement (ml/min/1.73 m**^**2)**^	0.68 (0.3–1.52)
**ID-urine volume (ml)**	750 (400–1000)

Data expressed as median (Q1, Q3) or frequency (%)

*Abbreviation*: *RKF* Residual kidney function, *ID* Interdialytic (44-hour)

The comparison of QOL domains according to RKF status between the two groups is summarized in Table 3. Patients with RKF had significantly higher scores for the symptom problem list (*p* = 0.009), cognitive function (*p* = 0.003), sleep (*p* = 0.011), overall heath (*p* < 0.001), physical functioning (*p* = 0.001), role limitations caused by physical health problems (*p* < 0.001), pain (*p* = 0.004), general heath (*p* = 0.001), role limitations caused by emotional health problems (*p* = 0.011), social function (*p* = 0.022), energy/fatigue (*p* = 0.011) and physical composite score (*p* < 0.001). As aforementioned in the methods section, high scores are associated with better quality of life.

**Table 3:** Quality of Life domains according to RKF status

QoL domains	Group A (RKF) (*n* = 29)	Group B (No RKF) (*n* = 49)	p
Symptom problem list	87.5 (77.08–91.67)	77.08 (69.7–87.5)	**0.009**
Effects of kidney disease	46.88 ± 19.2	46.88 ± 16.45	0.326
Burden of kidney disease	50 (31.25–68.75)	37.5 (12.5–65.62)	0.229
Cognitive function	86.67 (76.66–93.33)	73.33 (60–86.67)	**0.003**
Quality of social interaction	80 (70–93.33)	80 (66.67–93.33)	0.591
Sleep	80 (62.5–85)	55 (37.5–75)	**0.011**
Social support	66.67 (50–100)	83.33 (66.67–100)	0.621
Dialysis staff encouragement	62.5 (25–75)	62.5 (37.5–75)	0.646
Overall Health	70 (70–80)	60 (50–70)	**< 0.001**
Patient satisfaction	66.67 (50–66.67)	66.67 (50–66.67)	0.898
Physical functioning	75 (55–90)	45 (20–70)	**0.001**
role limitations caused by physical health problems	100 (50–100)	25 (0–62.5)	**> 0.001**
Pain	67.5 (48.75–77.5)	55 (22.5–67.5)	**0.004**
General health	35 (25–47.5)	25 (10–30)	**0.001**
Emotional well-being	60 ± 14.79	56 ± 15.27	0.379
role limitations caused by emotional health problems	100 (16.6–100)	0 (0–100)	**0.011**
Social function	75 (56.25–75)	37.5 (25–75)	**0.022**
Energy fatigue	55 (45–70)	35 (25–60)	**0.011**
Physical Composite Score	44.14 ± 9.47	34.24 ± 10.23	**< 0.001**
Mental Composite Score	46.20 ± 8.56	40.39 ± 8.84	0.111

*Abbreviation*: *QoL* Quality of life

Among the items of the MoCA score for cognitive function assessment, patients with RKF had significantly higher total score (*p* < 0.001), Visuospatial/ executive (*p* < 0.002), attention (*p* < 0.004) and delayed recall (*p* < 0.001) (Table 4).

**Table 4:** MoCA domains according to RKF status

MoCA domains	Max points of test	Group A (RKF) (*n* = 29)	Group B (No RKF) (*n* = 49)	*P*
Level of education (< 12 years)		23 (79.3%)	29 (57.1%)	**0.047**
Visuospatial executive	5	4 (3–5)	3 (1–4)	**0.002**
Naming	3	3 (3–3)	3 (3–3)	0.346
Attention	6	5 (4.50–5)	4 (4–5)	**0.004**
Language	3	3 (2–3)	2 (2–3)	**0.037**
Abstraction	2	2 (1.50–2)	2 (1–2)	0.237
Delayed recall	5	4 (3–4)	3 (3–4)	**0.001**
Orientation	6	6 (6–6)	6 (6–6)	0.407
The total score of MoCA	30	27 (26–28)	24 (19.5–26)	**< 0.001**

*Abbreviation*: *MoCA* Montreal Cognitive Assessment

Table 5 shows the correlation between the measured RKF and some demographic and clinical parameters. There was a statistically significant negative correlation between the measured RKF and hemodialysis vintage (*r* = − 0.529; *p* < 0.001). On the other hand, RKF had a significant positive correlation with both the PCS and total MoCA score (*r* = 0.365 and 0.47; *p* = 0.001 and < 0.001, respectively).

**Table 5:** Correlations of the measured residual kidney function

Variable	Correlation coefficient (r)	*P*
Age (years)	−0.121	0.292
Hemodialysis duration (years)	−0.529	**< 0.001**
Calcium (mg/dl)	0.009	0.935
Phosphorus (mg/dl)	−0.024	0.834
Parathyroid hormone (P.T.H) (pg/ml)	−0.128	0.263
Hemoglobin (g/dl)	−0.105	0.361
Ferritin (ng/ml)	−.035	0.760
TSAT (%)	−.066	0.565
KT/V	−0.058	0.617
physical composite score	0.365	**0.001**
mental composite score	0.170	0.137
The total score of MoCA	0.470	**< 0.001**

Multiple linear regression analyses were done to assess predictors of the measured RKF, the PCS and the MoCA score. The mean RKF and PCS were significantly decreased by 0.065 ml/min and 0.424, respectively for every 1-year increase in time since starting hemodialysis and the patient’s age, respectively. The mean MoCA score was significantly increased by 1.468 for every 1 ml/min increase in RKF. In addition, patient age was associated with inverse relation with the MoCA score. The mean MoCA score decreased by 0.14 for every 1-year increase in the patient’s age (Table 6).

**Table 6:** Predictors of RRF, physical composite and MoCA scores

Variable	B	Beta	95% CI	*P*
Residual kidney function (ml/min/1.73m^2^)				
Constant	0.69		(−1.47, 2.527)	0.456
Age (year)	−6.631	−0.001	(−0.012, 0.011)	0.99
Gender:				
Male	Reference			
Female	0.013	0.008	(−0.347, 0.373)	0.943
Diabetes mellitus	−0.139	−0.061	(− 0.659, 0.382)	0.596
Hypertension	0.128	0.062	(−0.347, 0.602)	0.593
HD duration (year)	−0.065	−0.295	(− 0.12, − 0.009)	**0.024**
HCV:				
Positive	Reference			
Negative	0.063	0.034	(−0.432, 0.558)	0.799
Physical composite score				
Constant	63.715		(49.407, 78.022)	< 0.001
Age (year)	−0.424	−.0646	(−0.538, −.31)	**< 0.001**
Gender:				0.095
Male	Reference	Reference	Reference	
Female	−3.206	−0.147	(−6.988, 0.576)	
Diabetes mellitus	−0.535	−0.019	(−5.53, 4.459)	0.831
Hypertension	1.274	0.040	(−4.207, 6.755)	0.644
RKF (ml/min/1.73m^2^)	2.337	0.165	(−0.15, 4.823)	0.065
HD duration (year)	−0.472	−0.153	(−1.029, 0.086)	0.096
MoCA Score				
Constant	33.556		(28.342, 38.769)	< 0.001
Age (year)	−0.14	−0.61	(−0.182, − 0.098)	**< 0.001**
Gender:				0.358
Male	Reference	Reference	Reference	
Female	−0.631	−0.083	(−1.989, 0.727)	
Diabetes mellitus	−0.078	−0.008	(−1.891, 1.735)	0.932
Hypertension	−0.901	−0.08	(−2.898, 1.096)	0.371
RKF (ml/min/1.73m^2^)	1.468	0.297	(0.603, 2.334)	**0.001**

*Abbreviation*: *HD* Hemodialysis, *HCV* Hepatitis C Virus, *MoCA* Montreal Cognitive Assessment, *RKF* Residual Kidney Function

## Discussion

Recent studies have concluded that the effects on improving the quality of life as well as on long-term survival should be considered when evaluating the effectiveness of the treatment in chronic diseases [22]. Also, it is increasingly recognized that renal failure is associated with cognitive impairment [23]. Among patients with CKD, the prevalence of mild cognitive impairment has been estimated to be as high as 30 to 63% as reported in studies by *Murray* et al. and *Post* et al.*,* respectively*,* which is approximately twice as high as in the age-matched general population [24, 25].

Residual Kidney Function is the remaining kidney function in patients receiving renal replacement therapy. RKF may provide many benefits to patients on permanent RRT. RKF preservation is expected to contribute significantly to the improvement of quality of life, cardiovascular protection, and even better survival in this patient population [26]. Therefore, this study was carried out to assess the impact of this issue on the quality of life and cognitive function in an Egyptian cohort of hemodialysis patients. There are recommendations for Residual Kidney function preservation as Avoidance of nephrotoxic agents, especially aminoglycosides, NSAIDs, CoX-2 (Cyclooxygenase-2) inhibitors and radiocontrast media, Avoidance of excessive ultrafiltration, routine use of biocompatible dialyzer membranes, routine use of bicarbonate- based dialysate, use of ultrapure water, hemodiafiltration, incremental hemodialysis, and a low-protein diet, as well as general care such as maintaining appropriate blood pressure, and better control of mineral and bone disorder parameters [27, 28].

As regards QOL domains according to RKF status, patients with RKF (urine output≥100 ml/day) had significantly higher scores (denoting better quality of life) for the symptom problem list, cognitive function, sleep, overall health, physical functioning, role limitations caused by physical health problems, pain, general health, role limitations caused by emotional health problems, social function, energy/fatigue and PCS in comparison with patients without RKF. Moreover, the measured RKF was positively correlated with the PCS. These findings match with the results of *Abdel-Azim* et al., who revealed that HD patients with preserved RKF had better physical functioning, role limitations caused by physical health problems, vitality, mental health, general health, PCS and MCS scores. Also, they observed that there was a statistically significant positive correlation between measured RKF and PCS, Symptom problem list, cognitive function, sleep, overall health, physical functioning, role limitations caused by physical health problems, pain, general health, role limitations caused by emotional health problems, social function and energy/fatigue scores [29]. In addition, *Hiramatsu* et al. observed that preservation of urine volume showed a positive relationship with physical activity [30]. However, when subjecting possible predictors of PCS to multivariate regression analysis, patients’ age was the only predictor for PCS. Such a result goes in agreement with, *Seica* et al. who demonstrated that age had a significant impact on HRQOL especially PCS of the SF-36, but not on MCS [31].

Sleep disturbances are widespread among HD patients, and they are frequently linked to pain, exhaustion, and sadness [32]. Sleep disturbances were shown to be more common in HD patients lacking RKF in the current research. Psychological disorders, pulmonary edema from fluid overload, disturbances in the activity of the respiratory center from chronic metabolic acidosis and uremic toxins, abnormalities in dopaminergic pathways, anemia, and increases in calcium-phosphate product and PTH levels are all possible causes of sleep disorders in HD patients [33].

Cognitive impairment is a highly relevant clinical factor for disease progression in HD patients, possibly also affecting daily life activities, thereby impeding adherence to therapeutic regimes and compromising the quality of life [34]*.* It was evident that cognitive impairment is more prevalent in individuals with CKD than in the general population [35].

As regards cognitive function domains according to RKF status, visuospatial, executive, attention, language, delayed recall and total score of MoCA were significantly higher in the RKF group compared to the non-RKF group. Also, there was a statistically significant positive correlation between the measured RKF and visuospatial, executive, attention, delayed recall and the total MoCA score. Moreover, the measured RKF, were proved to be one of the predictors of the total MoCA score by multivariate linear regression analysis. To the best of our knowledge, there are no available studies in the literature that had assessed the relation between MoCA score and RKF status. Most of the studies had assessed the relation between cognitive function and CKD and ESKD patients in general.

This study had limitations. First, a relatively small number of patients were studied. Second, the cross-sectional nature of the study. Third, absence of calculated Charlson or another comorbidity scores. However, assessing the relation between the measured residual kidney function and both the cognitive function and HRQOL in this specific group of patients is considered as a strength point in the current study.

## Conclusion

The duration of hemodialysis appears to diminish residual kidney function. When compared to patients without RKF, HD patients with maintained RKF had a better quality of life and cognitive function, however, the patients’ age should be taken into account. The relevance of RKF regular monitoring and preservation in HD patients is highlighted by these findings.

## Data Availability

All data analyzed during the current study available from the corresponding author on reasonable request.
